# Therapeutical Notes

**Published:** 1899-02-11

**Authors:** 


### THERAPEUTICAL NOTES.

### Mucous Colitis.

W. H. Porter, writing in the *Post Graduate*, September, 1898, recommends tanalbin for this troublesome affection in doses of 5 to 15 grains three times daily. A diet composed of skim milk or butter milk only may be employed with advantage in some of these cases.

